# Supplementary material for: Hidden network preserved in Slide-tags data allows reference-free spatial reconstruction
Source: Nat Commun. 2025 Oct 31;16:9652. doi: 10.1038/s41467-025-65295-w (PMC12579210; doi:10.1038/s41467-025-65295-w)
Supplement: Supplementary file 1 — Supplementary Figs. [file 41467_2025_65295_MOESM1_ESM.pdf]

Hidden network preserved in Slide-tags data allows reference-free spatial reconstruction

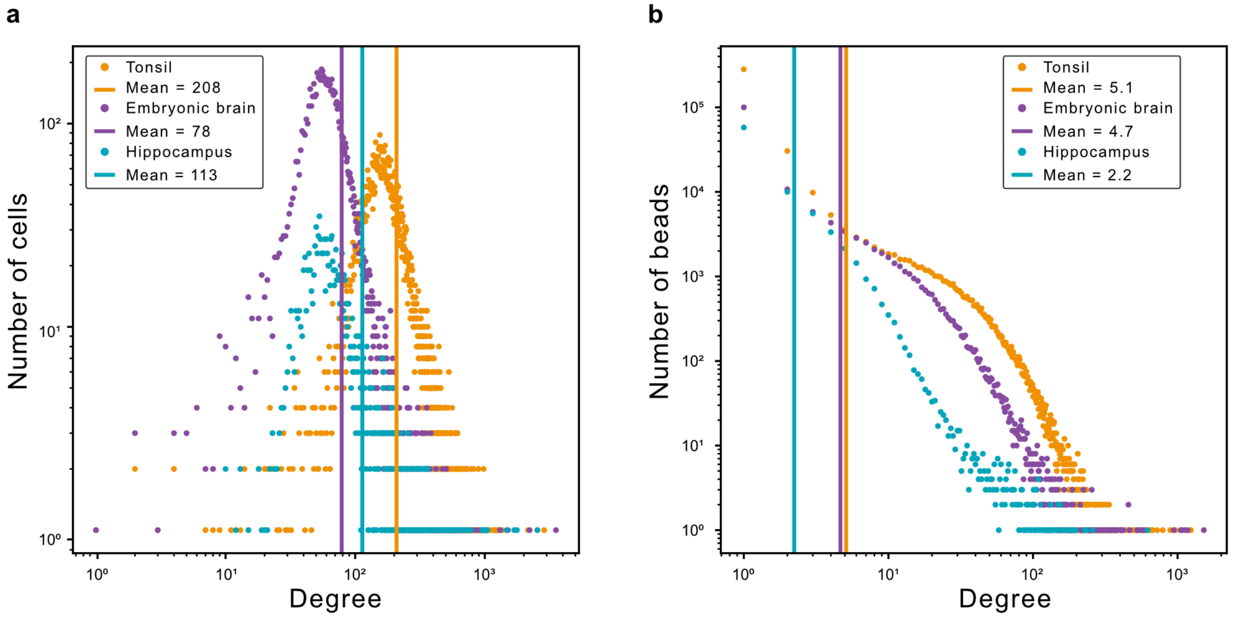

Supplementary figure 1. Degree distributions for all samples (a) Degree distributions for all the cell-nodes in each sample network, showing shifts between samples in the number of connections that a given cell has, with the tonsil samples exhibiting a distribution peaking at a higher degree than the other two samples. **(b)** Degree distributions for all the bead-nodes in each sample network. The three plots display fundamental differences in network characteristics, with the mouse hippocampus sample exhibiting a power (linear on log-log scale) relationship that might be typical of scale-free or preferential attachment networks, whereas embryonic brain and tonsil samples show an apparent combination of a power relationship (linear on log-log) with a distinct upward bulge at intermediate degrees, suggesting a mixture of two underlying processes: a heavy tailed component consistent with preferential attachment dynamics and a finitely distributed component reflecting spatially constrained or homogeneous edge formation. Source data are provided as a Source Data file.

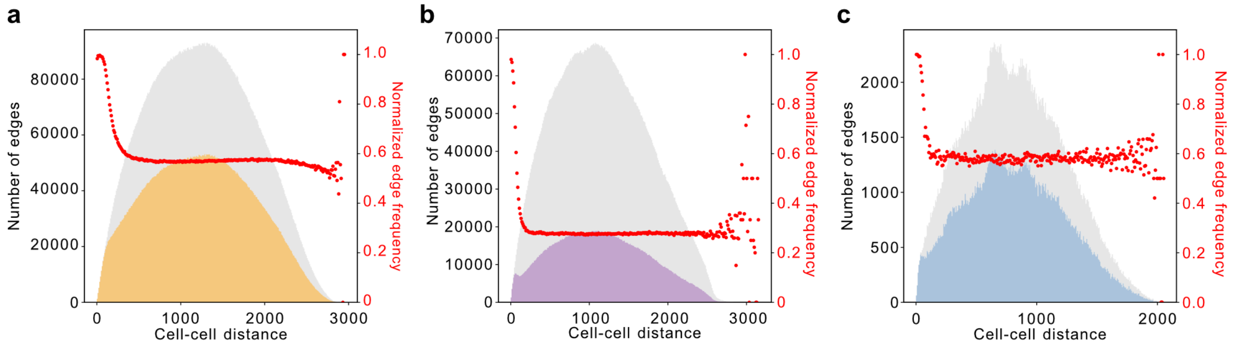

Supplementary figure 2. Unipartite edge length distributions (a) Distribution of cell-cell edge lengths in the tonsil network (orange), compared to a theoretical spatial null model or baseline distribution (gray) in which all pairwise cell-cell distances are sampled uniformly within the tissue geometry. Deviations from this distribution reveal enrichment or depletion of connections at different length scales in the observed network. The red line shows the ratio of observed to the null model. **(b)** Equivalent analysis for the mouse embryonic brain sample (purple). **(c)** Equivalent analysis for the mouse hippocampus sample (blue).

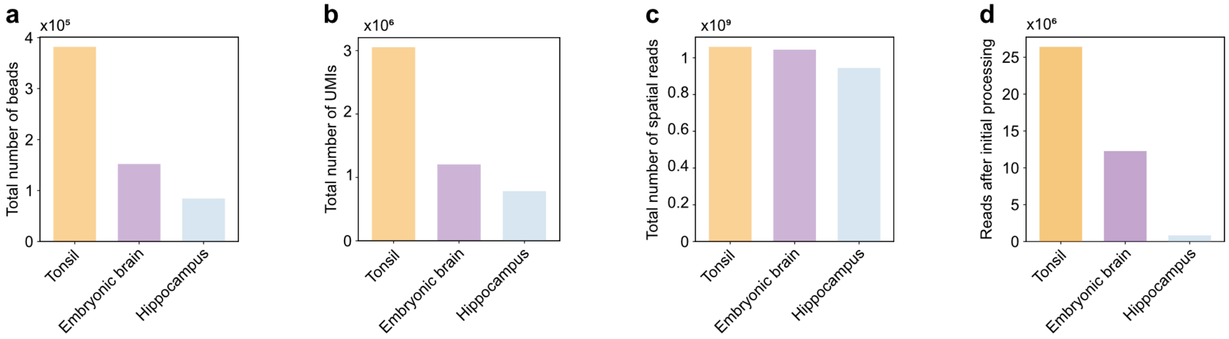

Supplementary figure 3. Sample-specific properties and sequencing metrics (a) Number of beads in each network for each respective sample **(b)** Number of total UMIs for each respective sample network, before any filtering or further processing. **(c)** Number of raw reads before any processing for each respective sample, notably each sample has almost the same number of reads. **(d)** Number of reads retained after the initial Slide-tags sequence pre-processing step, reflecting the yield of usable reads in each dataset. Source data are provided as a Source Data file.

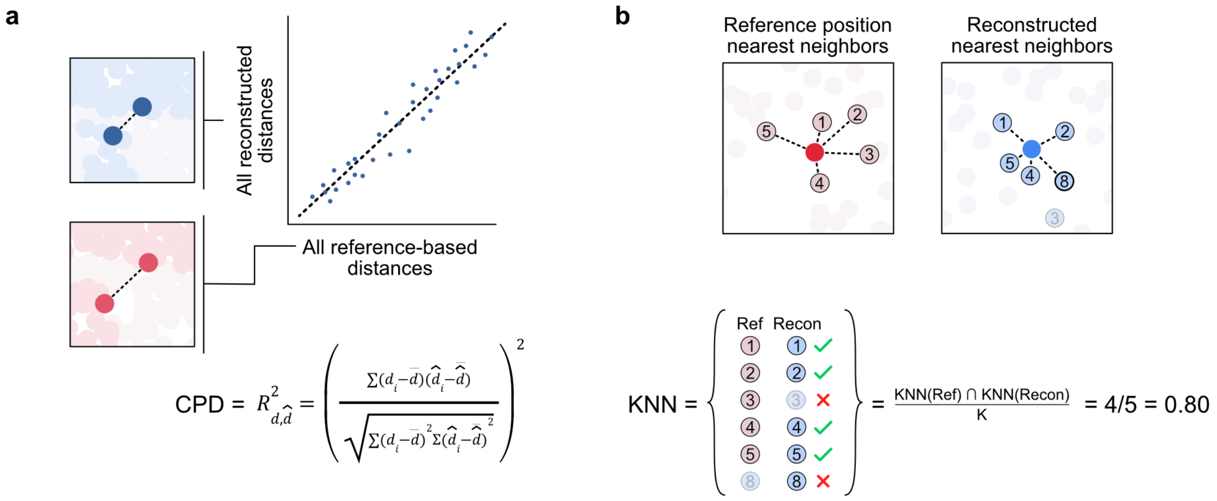

Supplemental figure 4. Quality metrics for assessing network-based spatial reconstruction fidelity. (a) Schematic illustration of metric for quantifying global reconstruction fidelity using the pairwise correlation between the set of Euclidean distances between each nodes to all other nodes for the reference and reconstructed sets of spatial positions, where the score is the square of the correlation coefficient. **(b)** Schematic illustration for quantifying the local reconstruction fidelity by using the overlap in identities of the *k* closest nodes between the reference positions and the reconstructed positions, with the score for the reconstruction as a whole taken as the mean for all nodes.

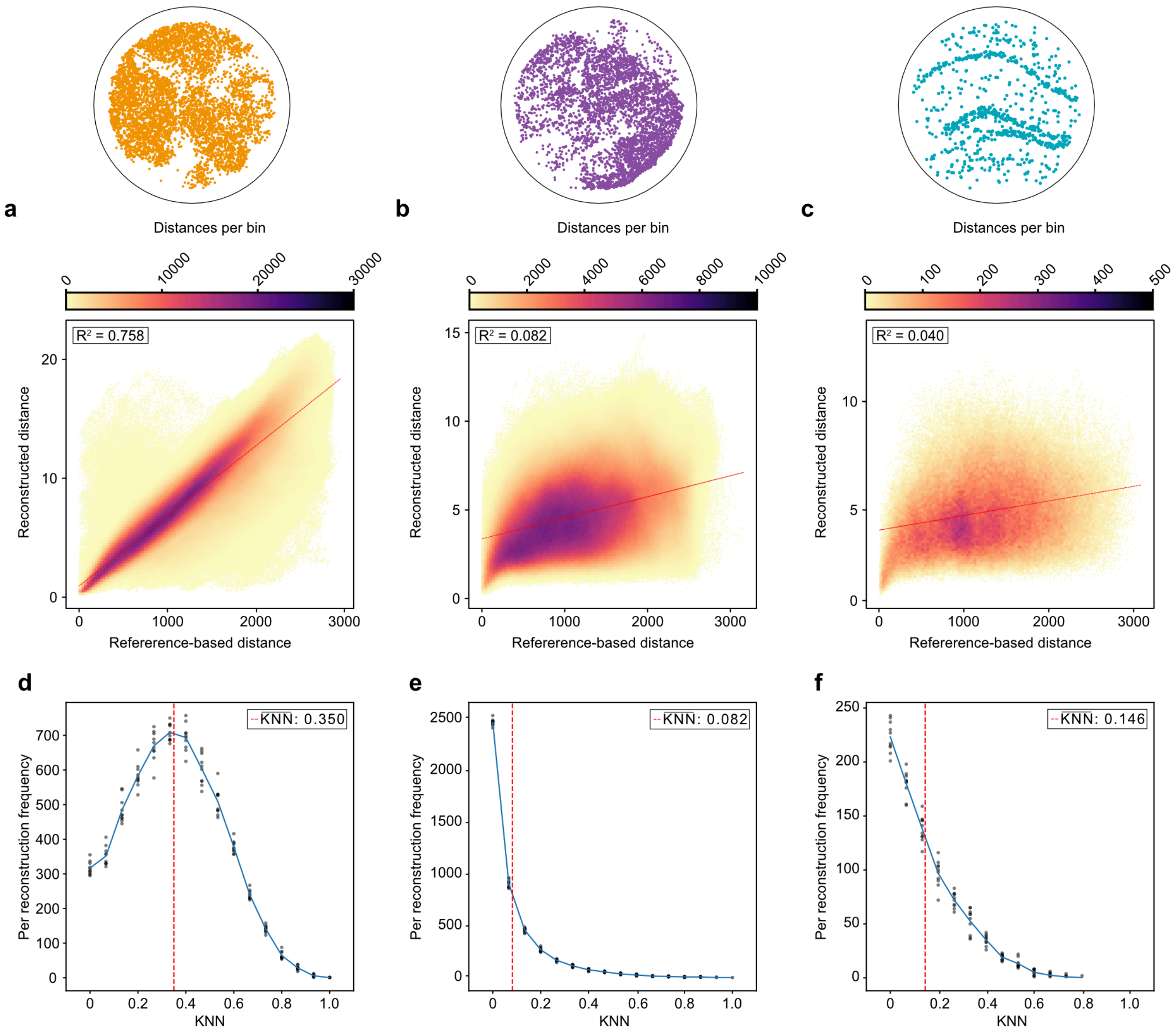

Supplementary figure 5. Distributions for the CPD quality metric for each sample for base reconstruction. Scatter-plots of mean reconstructed distance between any two nodes in the sample (over 10 reconstructions) compared with corresponding pairwise inter-node distances in the reference distance (coloured by point density) for the **(a)** tonsil sample **(b)** embryonic sample **(c)** hippocampus sample. Frequency count of the KNN values for all cells for each 10 reconstructions as points, with each reconstruction as a point per value and the mean drawn as a line for the **(d)** tonsil sample **(e)** embryonic sample **(f)** hippocampus sample.

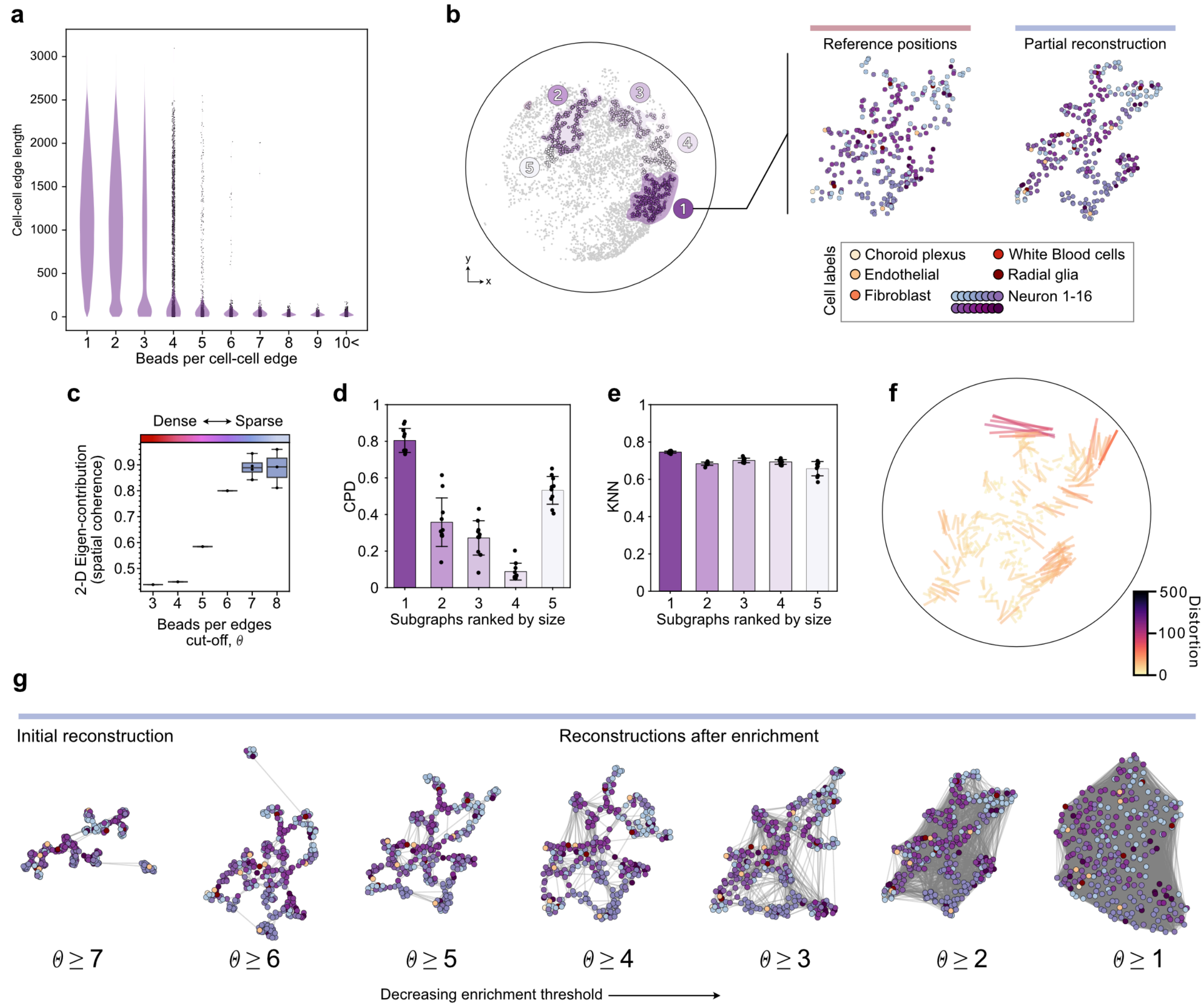

Supplementary figure 6. Reconstruction of partial networks possible with the lower quality of the spatial networks generated by the mouse embryonic sample (a) Cell-cell edge length visualized as the number of bead barcodes which the edge consists of increases, the distribution of edge lengths decreases, going from mainly being composed of long-range, seemingly random edges to mainly consisting of short-range, spatially informative edges. **(b)** The evolution of Gram matrix spatial coherence as a function of filtering stringency, or the number of beads per edge. **(c)** Highlighting of the 5 largest subgraph extracted from the mouse embryonic sample which can be reconstructed individually, in addition to the reconstruction of the largest such region of 286 cells **(d)** Reconstruction qualities for all the partial reconstructions, both global (left) and local (right). **(e)** Showcasing the process of resampling edges within sparser networks to generate a reconstruction of higher global fidelity. **(f)** Reconstruction compared to reference cell positions for the largest subgraph with distortion lines to show relative positioning error of each cell. **(g)** Progression of network-based spatial reconstruction of the 286 cell subgraph identified under stringent filtering conditions as the filter is relaxed to add edges to internal (within subgraph) cell nodes. I.e. no external connections are added, so the subgraph number of nodes remains the same. Spatial reconstructions improve up to a point and then deteriorate as the graph becomes fully connected at the limit of zero filtering threshold. Data in bar graphs are presented as mean ± SD over N = 10 reconstructions of the network relevant to each respective bar, characterizing technical variance in STRND reconstruction. Source data are provided as a Source Data file.

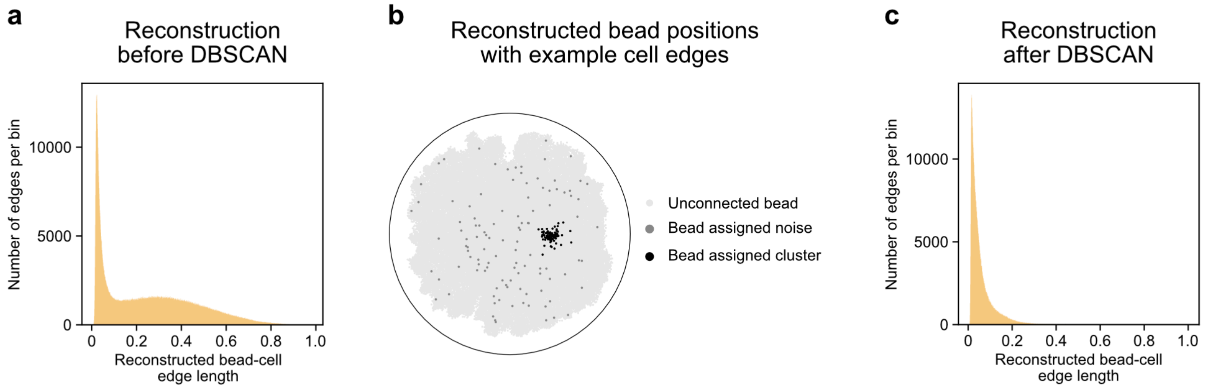

Supplementary figure 7. Iterative reconstruction process by DBSCAN clustering (a) Distribution of bead-cell edge lengths after initial reconstruction, showing what appears to be a bimodal distribution likely consisting of spatially generated edges and noise. **(b)** Bead positions for an example cell with all reconstructed bead positions (light gray), beads not attributed to a cluster (dark (gray) and cluster of beads as calculated by DBSCAN (black) chosen by its clear distinction between clustered and non-clustered beads. **(c)** Distribution of bead-cell edge lengths after DBSCAN-based edge removal and an additional reconstruction, showing what appears to be a unimodal distribution.

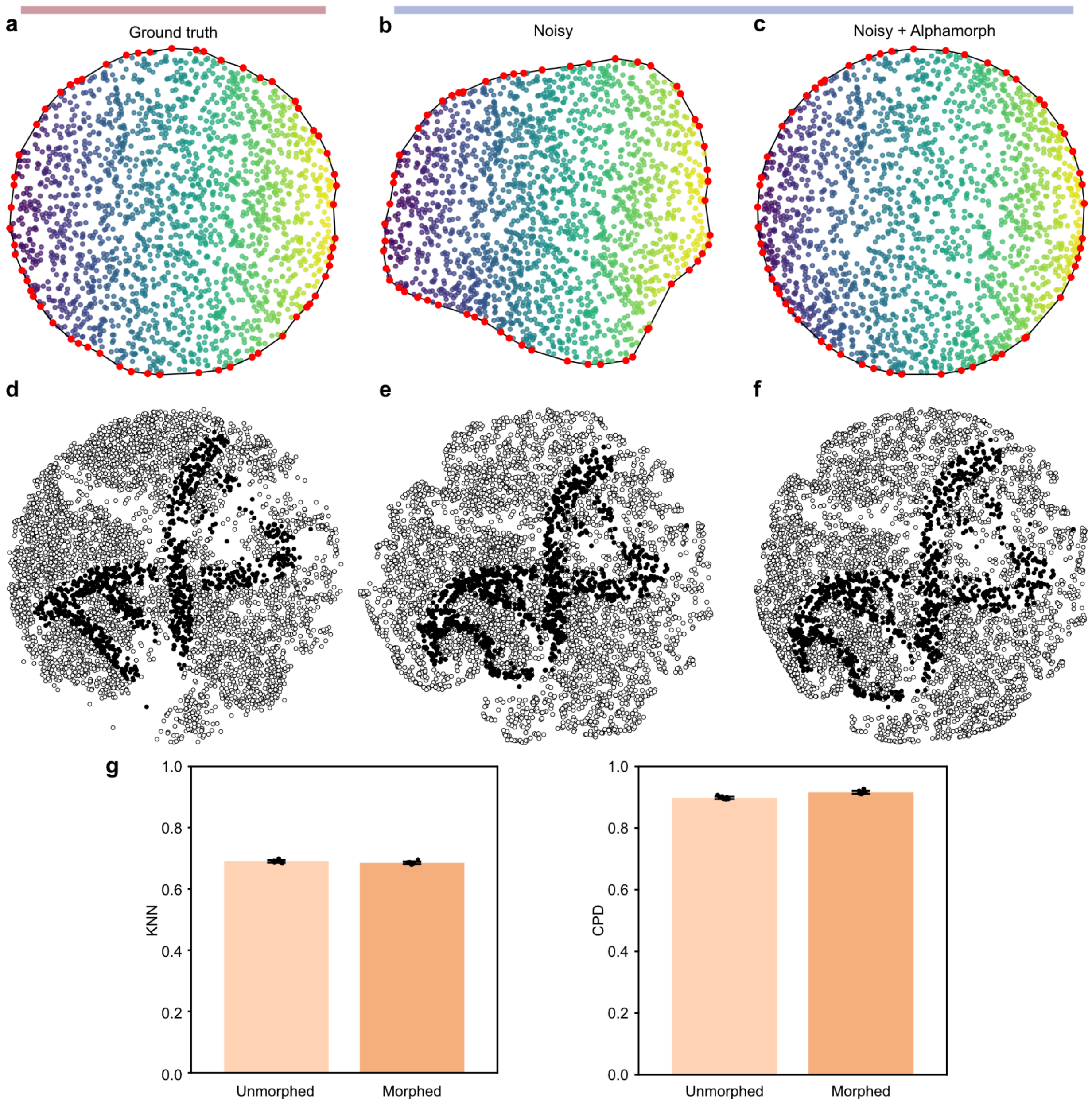

Supplementary figure 8. Application of shape morphing to improve global geometric structure in reconstructed point clouds. (a) A synthetic point cloud with a well-defined circular shape used as ground truth. Red points indicate alpha shape boundary landmarks automatically detected from the outermost points. **(b)** A perturbed version of the point cloud, where noise distorts the boundary shape. Red points indicate source landmarks. **(c)** The reconstructed point cloud after applying *Alphamorph*, which corrects the boundary back to circular geometry. Here, red points indicate target landmarks used for shape correction. **(d)** Reference positions for the iteratively reconstructed tonsil network with each point colored by image sampling. **(e)** The iteratively reconstructed tonsil network before applying *Alphamorph.* **(f)** *Alphamorph* applied to the iteratively reconstructed tonsil network **(g)** Bar plots showing change in reconstruction quality metrics before and after shape morphing. Data in bar graphs are presented as mean ± SD over N = 10 reconstructions of the network relevant to each respective bar, characterizing technical variance in STRND reconstruction.

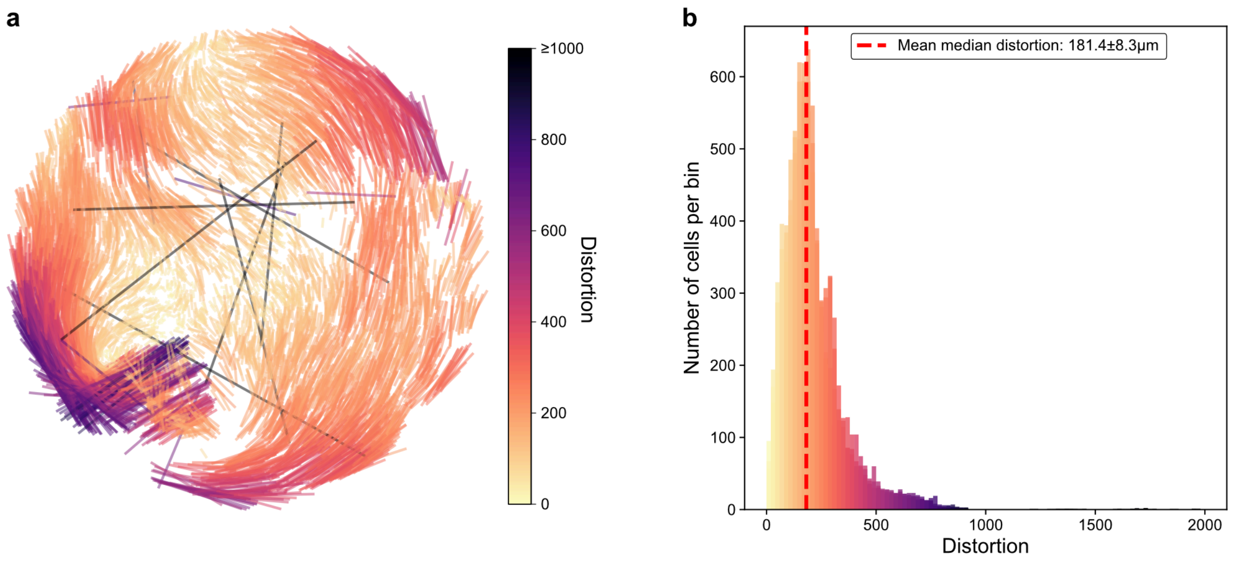

Supplemental figure 9. Distortion of the reference cells in the iteratively reconstructed tonsil sample (a) The change in positions after performing an alignment between reconstructed points to the reference positions. **(b)** Distribution of distortion lengths for N = 10 reconstructions, with the mean value of the median reported over all reconstructions reported. Distortion lengths are asymmetrically distributed, with a majority appearing close to the median while a long tail of small highly distorted regions exists. Source data are provided as a Source Data file.

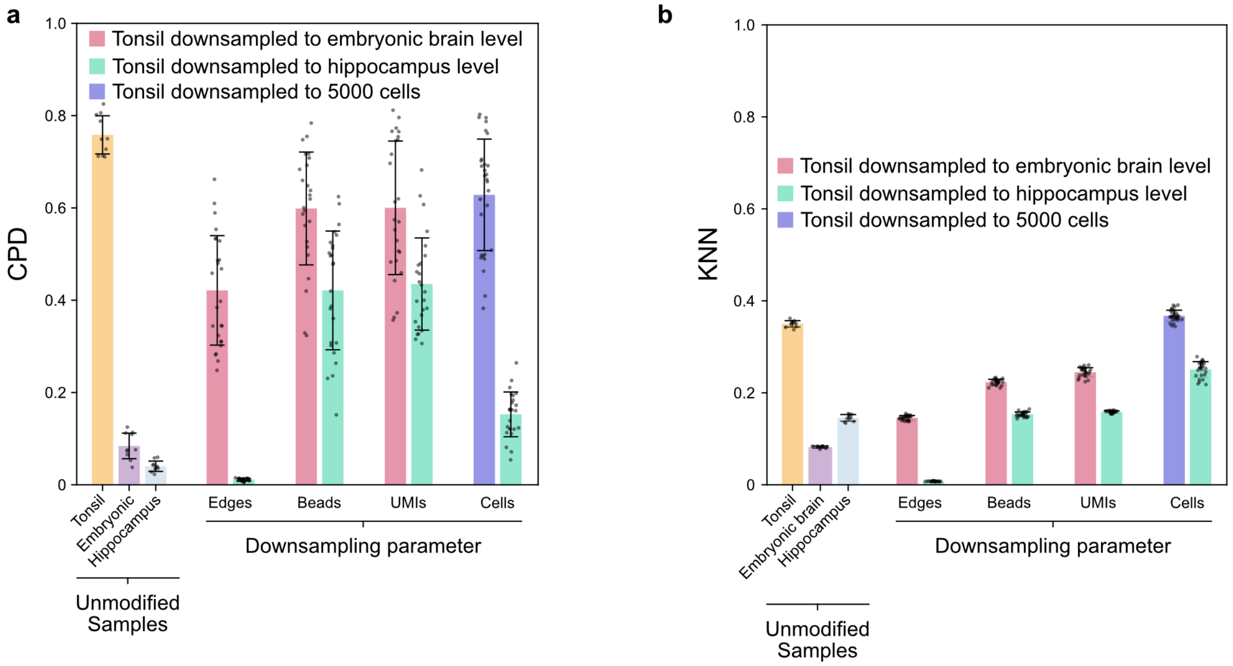

Supplementary figure 10. Mouse full network reconstructions compared to downsampled tonsil network (a) Global quality metrics of the tonsil sample after random downsampling to the the level of the each mouse sample with respective to the number of edges, beads, and UMIs, with cells being downsampled to 5000 cells instead of embryonic brain network due to similarity in cell amounts showing how the edge downsampling having the most significant impact with the hippocampus level downsampling reconstructing worse than the hippocampus sample, and the other factors having comparatively minor effects on global reconstruction quality albeit with a large variance between individual reconstructions and downsamplings. **(b)** Local quality metrics for the same downsampled networks, showing minor effects on local reconstruction quality, with the sample downsampled 5000 cells even showing a slight improvement over the unmodified network. Data in downsampled bars are presented as mean ± SD, where SD is calculated through taking the square of the sum of the variances for each downsampled network. Variance in downsampling was assessed by performing it 5 times with separate random seed, and variance in STRND reconstruction was by reconstructing each downsampled network 5 times for a total of N=25. The unmodified samples are identical to the samples present in Figure 3b. Data in bar graphs are presented as mean ± SD over N = 5 reconstructions for N = 5 independent downsamplings for a total of N = 25 of the network relevant to each respective bar, characterizing technical variance in STRND reconstruction and the random downsampling. Source data are provided as a Source Data file.

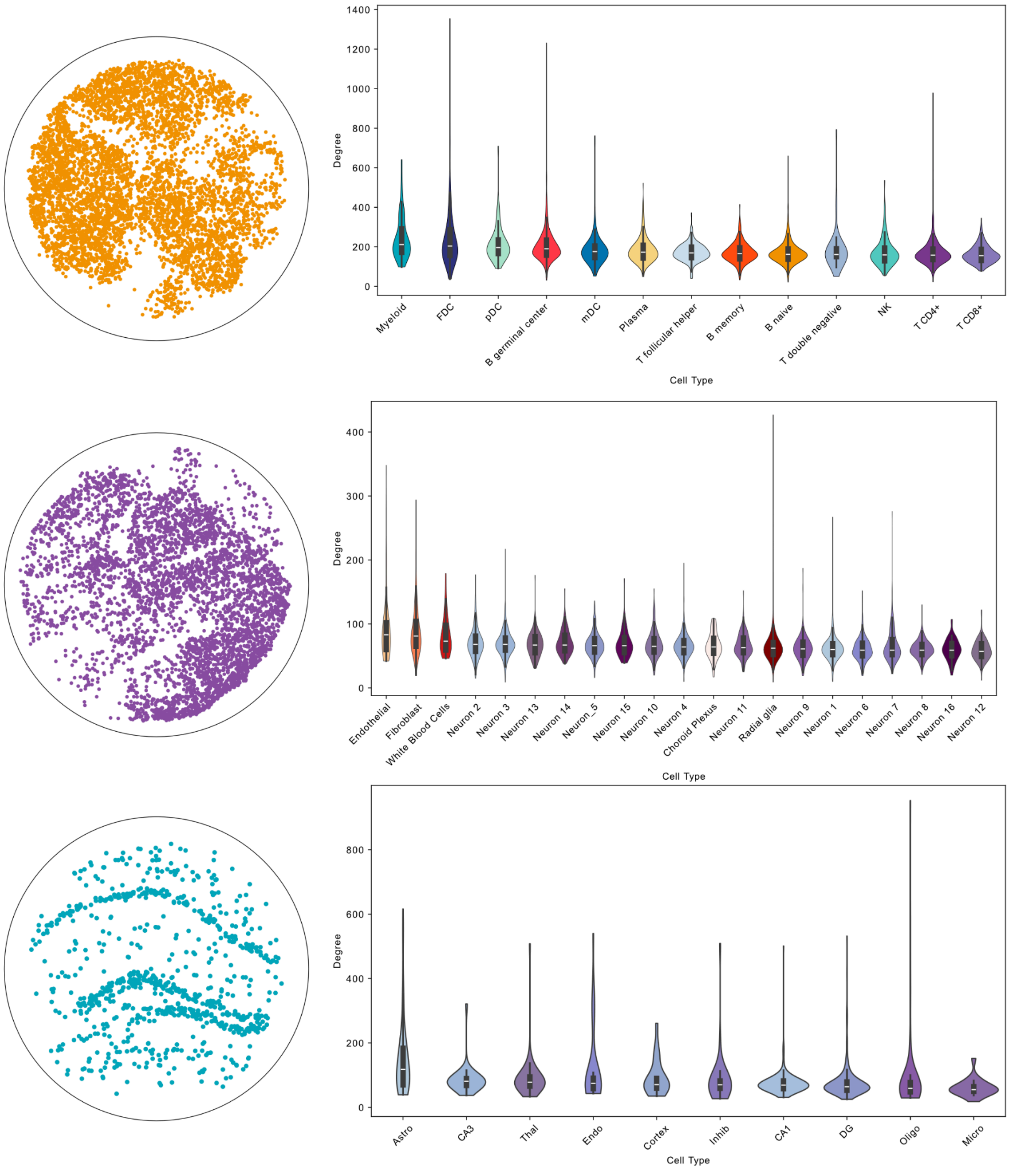

Supplementary figure 11. Degree distributions for each cell type for each sample. Violin plots showing the distribution of node degrees (number of cell-bead associations) of individual cells, grouped by Russel et al. annotated cell type, across three tissue samples: human tonsil (top), mouse embryonic brain (middle), and mouse hippocampus (bottom). The distributions show both tissue-specific differences in labeling density and variability in network structure, as well as differences among cell types. All boxes within violin plots present median, upper and lower quartiles, with whiskers showing 1.5× interquartile range

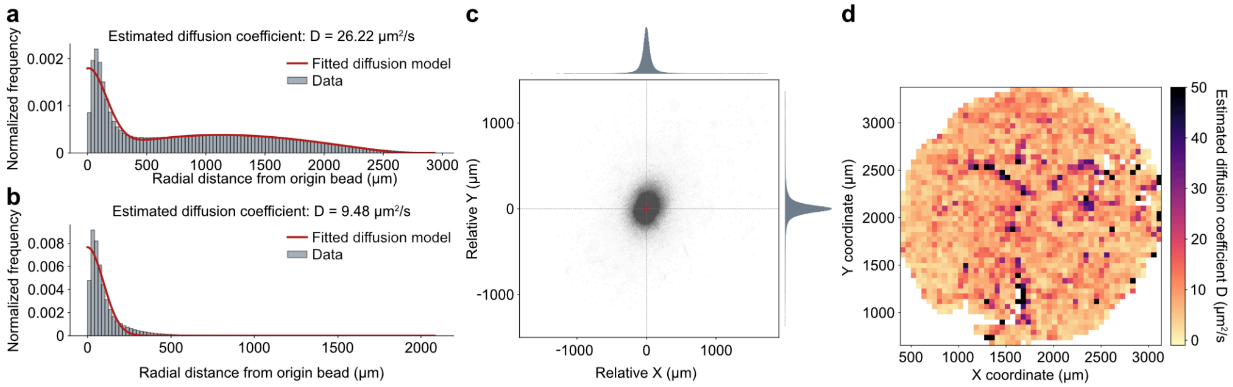

Supplementary figure 12. Estimated diffusion profile for an average bead estimated using final tonsil reconstruction. (**a**) Radial histogram of distances between beads and their associated nuclei, pooled across the full network. The bars represent normalized frequency of observed distances. The red curve is the best-fit model of a 2D Fickian diffusion profile with a single characteristic diffusion coefficient D superimposed with the distribution of random line segments in a disc. (**b**) Radial histogram and curve fit after DBSCAN-filtering. (**c**) 2D diffusion profile shown as a scatter plot of the reconstructed relative coordinates of all cell nuclei associated with a single origin bead (from the iterative reconstruction), shown in coordinates centered around each bead (bead origin - red). Each black dot represents a cell tagged by the bead's barcode, revealing the local spread pattern of barcodes during diffusion. (**d**) Spatial heatmap of inferred diffusion coefficients across the reconstructed sample. Each bead with a fitted diffusion coefficient was assigned to a grid bin in a 170x170 spatial array spanning the minimum and maximum reconstructed coordinates.

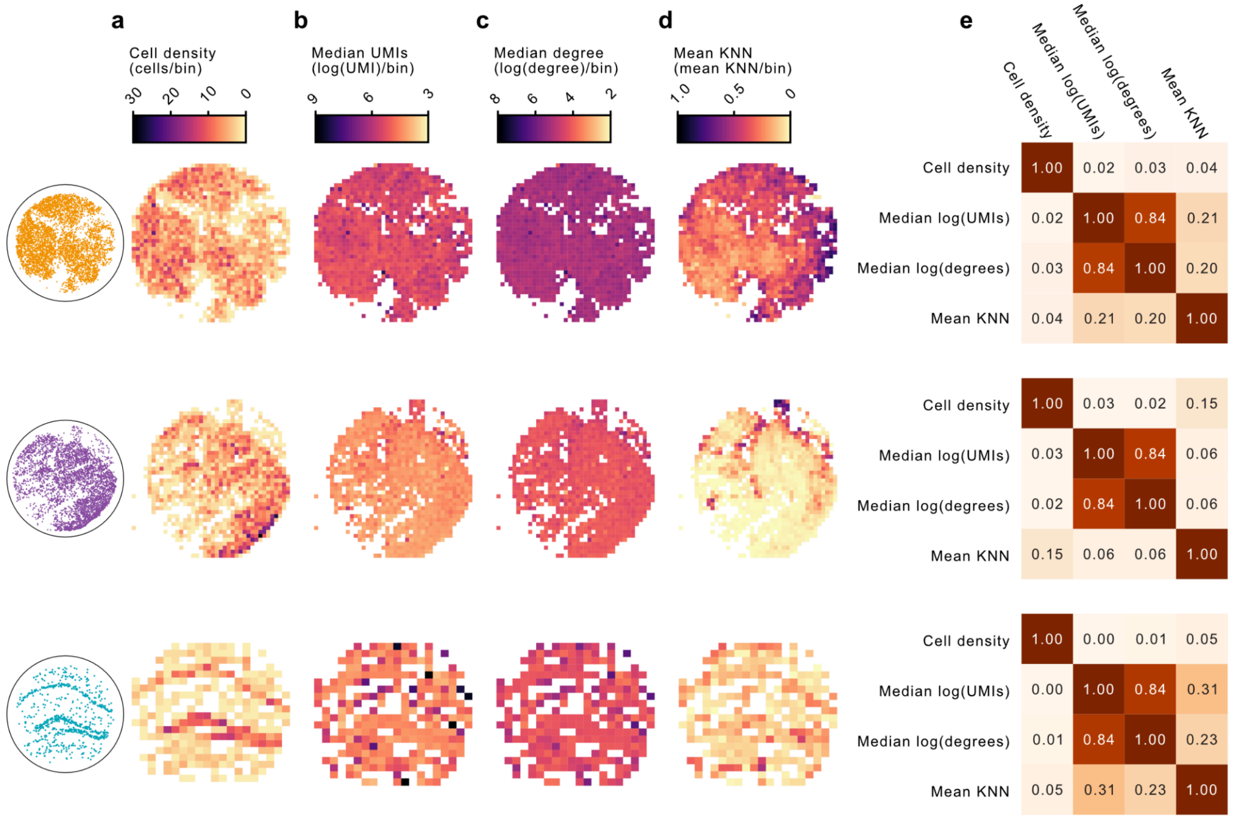

Supplementary figure 13. Relationships between sample properties. (**a**) Binned plot of the reference positions for each sample where each bin is coloured by the number of cells in each bin. (**b**) Bins coloured by the log of the median UMIs in each bin (**c**) Bins coloured by the log of the median degree in each bin (**d**) Bins coloured by the mean KNN score in each bin for the initial network (**e**) Pairwise Spearman correlation values for all different combinations of properties using the values of the bins.

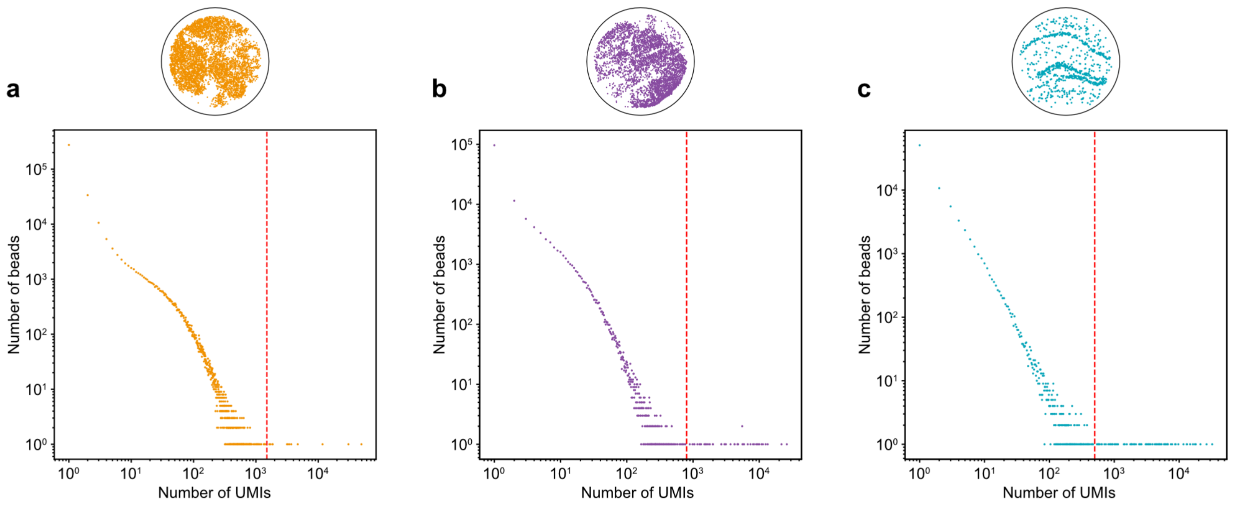

Supplementary figure 14. UMI distribution of the beads across samples Log-log plots showing the distribution of total UMI counts per bead for (**a**) human tonsil (**b**) mouse embryonic brain (**c**) mouse hippocampus. The horizontal axis shows the total UMI count per bead, and the y-axis shows the number of beads with that UMI count on log scale. The red-dashed line indicates the minimum UMI threshold used for filtering beads prior to network reconstruction. These plots show the heavy-tailed nature of the UMI distribution and presence of a low-UMI population that was excluded to reduce noise and improve reconstruction quality.

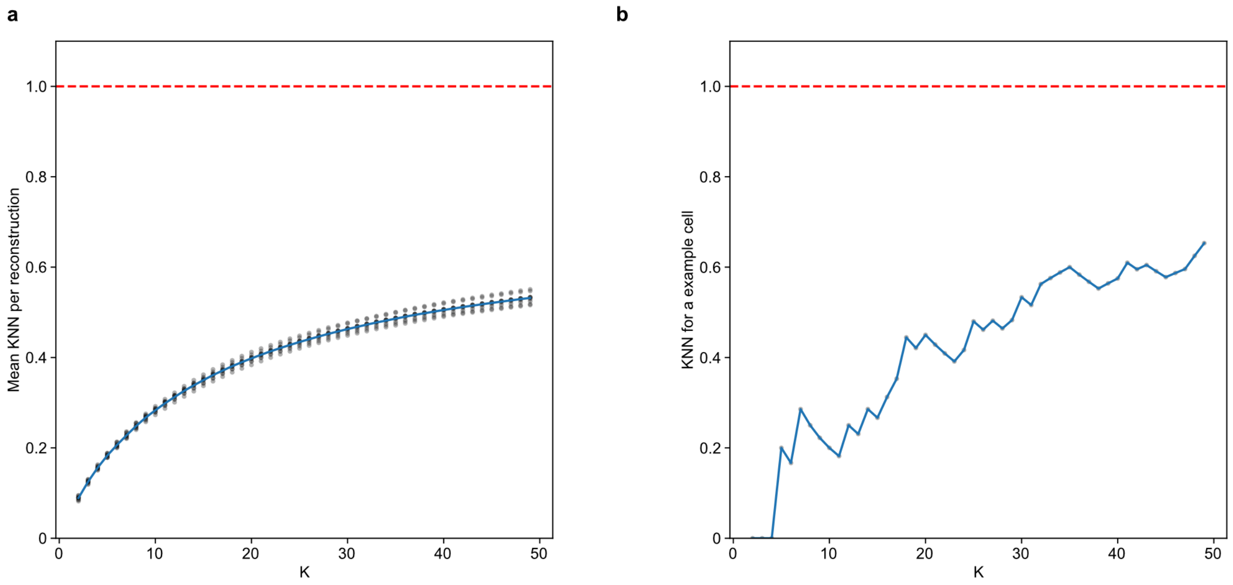

Supplementary figure 15. Dependence of KNN score on neighborhood size *k* at global and single-node levels (a) Mean KNN score across all nodes as a function of neighborhood size *k*, computed for each of 10 independent reconstructions (points), with the average across runs shown as a line. **(b)** Plot showing how the KNN for a single node from a representative reconstruction varies as *k* increases, in contrast to the strict increase for the entirety of reconstruction visible in (a). The red dashed line in both plots indicates the theoretical maximum KNN score of 1.0. These plots show that global neighborhood preservation improves with larger *k*, while individual nodes may vary in local preservation.

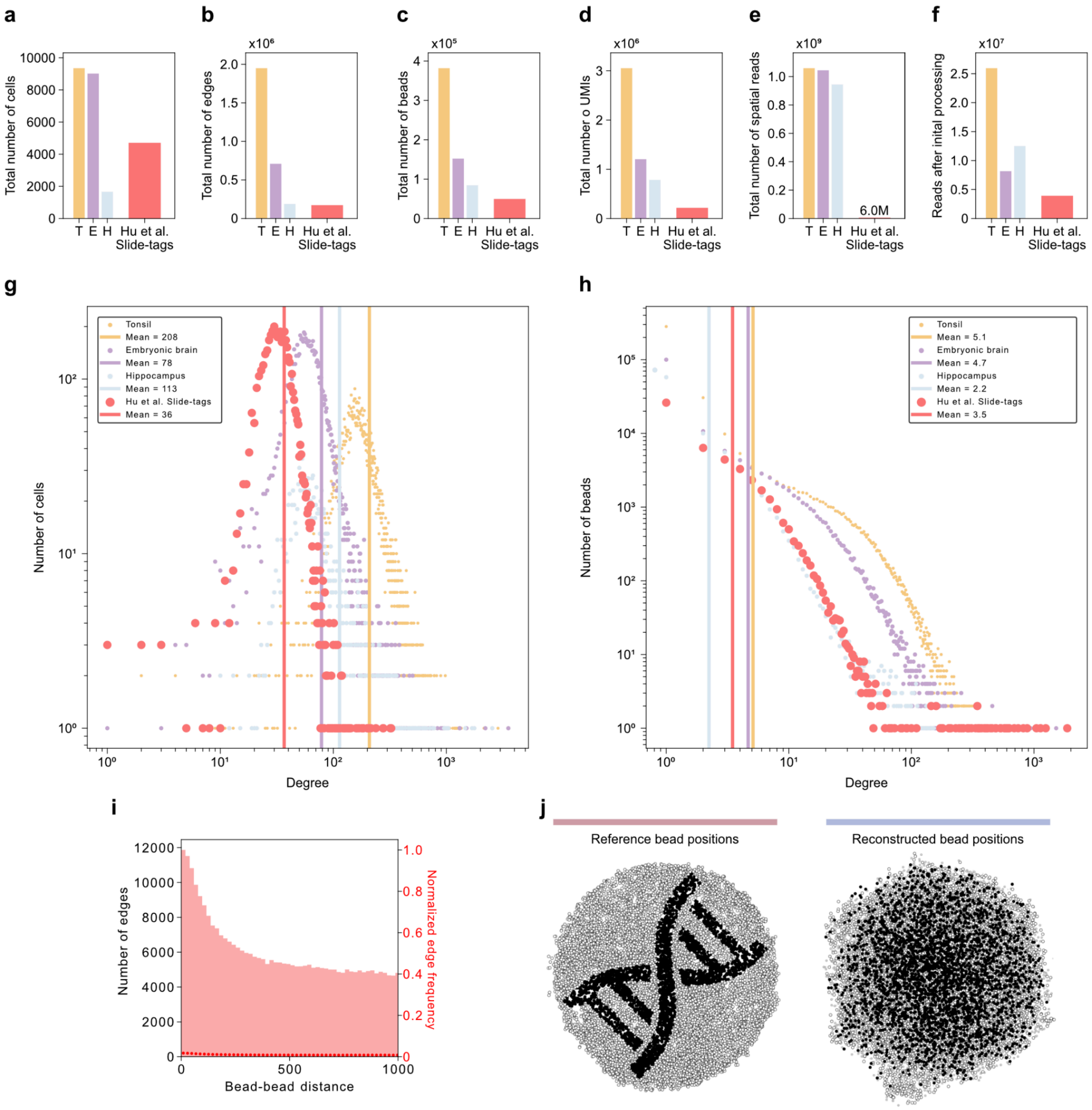

Supplementary figure 16. Network properties and initial reconstruction of the slide-tags data of Hu et al. Basic network and sample parameters of the Hu et al. network compared to the Russell et al. tonsil (T), mouse embryonic brain (E), and mouse hippocampus (H) samples in regards to (**a**) number of cells, (**b**) number of edges, (**c**) number of beads, (**d**) number of UMIs, (**e**) total number of reads, and (**f**) number of reads after initial processing showing it is overall lower, with especially the number of raw reads being almost three orders of magnitude lower but still containing a large fraction of correct reads. (**g**) Degree distribution of the cells of the Hu et al. sample highlighted, showing that overall each cell has fewer edges than any of the other samples. (**h**) Degree distribution of the beads of the Hu et al. sample showing a similar distribution to the mouse hippocampus sample. (**i**) Distribution of bead-bead edge lengths in the Hu et al. network unipartite projection, revealing an enrichment of short edges. (**j**) Reconstruction of the Hu et al. sample network with an image overlaid on the reference bead positions showing how the network does not generate a high-quality reconstruction based purely on bead-cell edges..

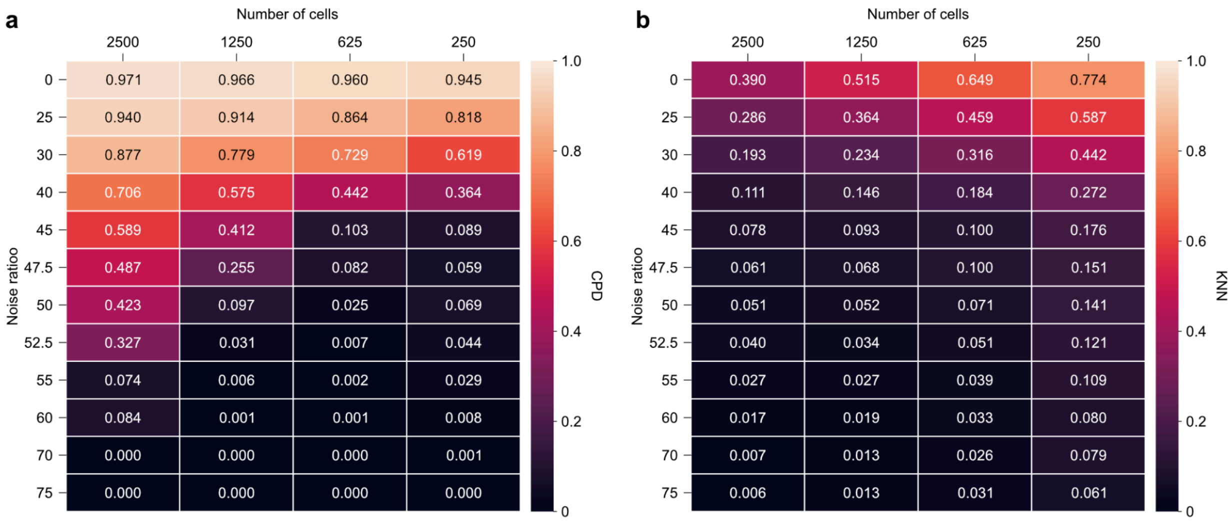

Supplementary figure 17. Heatmap of reconstruction quality metrics of simulated data showing effect of cell density and noise on reconstruction quality. (**a**) Heatmap showing how the global reconstruction quality metric changes as the cell density and noise ratio is varied, showing how less dense networks appear more sensitive to noise, in addition to the rapid changes in quality that can occur across a certain threshold, with the threshold varying based on network density. (**b**) Heatmap showing how the local reconstruction quality metric changes as the cell density and noise ratio is varied, showing how the less dense networks have a higher local quality which indicates that less dense network, while still noise susceptible, should better reconstruct exact neighbourhoods of reconstructed networks likely due to slight changes in reconstructed position having less effect on the rank of each cells neighbors. Shared simulation conditions for all instances are diffusion coefficient = 10, diffusion time = 480s, starting number of beads = 25000, and area circular diameter = 1500 mm. Source data are provided as a Source Data file.
